# Blood Donation Deferral at a Tertiary Centre in Riyadh: Epidemiology, Sex-Specific Patterns, and Donor Retention Outcomes

**DOI:** 10.3390/jcm15176859

**Published:** 2026-09-04

**Authors:** Nawaf Oraijah, Muhammad Raihan Sajid, Rimah Abdullah Saleem, Jana H. Husein, Hibba Siraj, Salman Aldosari, Ammar Alsughayir, Mohammed Alkhalifah, Naif Bin Muhanna, Sara Bakr Abobakr, Alyazeed Alsaif

**Affiliations:** 1 College of Medicine, Alfaisal University, Riyadh 11533, Saudi Arabia; 2 Department of Pathology, College of Medicine, Alfaisal University, Riyadh 11533, Saudi Arabia; 3 Department of Biochemistry and Molecular Medicine, College of Medicine, Alfaisal University, Riyadh 11533, Saudi Arabia; 4 Pathology and Clinical Laboratory Medicine Administration, Blood Bank and Transfusion Medicine Services Department, King Fahad Medical City, Riyadh 11525, Saudi Arabia

**Keywords:** blood donation, donor deferral, transfusion-transmitted infections, hemoglobin, vital signs, blood bank, Saudi Arabia, donor retention

## Abstract

**Background**: Blood donation deferral poses a significant challenge to transfusion services worldwide, threatening blood supply adequacy and donor retention. Population-specific data on deferral epidemiology and subsequent donor return behavior in Saudi Arabia remain limited. This study aimed to characterize the prevalence, reasons, and demographic patterns of blood donation deferral at King Fahad Medical City (KFMC)—a major quaternary referral center in Riyadh—and to quantify the rate at which temporarily deferred donors re-engage with the donation process at the encounter level. **Methods**: A retrospective observational study was conducted on records of all registered volunteer blood donor encounters at KFMC from 1 January 2023 to 31 December 2024 (*n* = 60,290), extracted from the Hematos IIG blood bank information system. Donor demographics (age, sex, weight, nationality), deferral classification (temporary, permanent, indeterminate), deferral reason, pre-deferral donation frequency, and post-temporary-deferral donation frequency were analyzed. Inferential statistics employed the Chi-square test and Fisher’s exact test (significance threshold: *p* < 0.05). A multinomial logistic regression, restricted to the 10,681 encounters classified as temporarily deferred, identified correlates of post-deferral donation frequency, adjusted for donation frequency before deferral. **Results**: Of 60,290 registered donor encounters, 18,458 (30.6%) resulted in deferral. Male donors constituted 86.4% of deferrals; the 31–45-year age group accounted for 47.3%. The three leading deferral reasons were abnormal vital signs (39.4%), post-donation transfusion-transmitted infectious disease (TTID) screening reactivity (35.6%), and low hemoglobin (14.0%). Sex-stratified analysis revealed that among female donors, low hemoglobin (40.50%) exceeded abnormal vital signs (35.50%) as the leading reason. Among the 10,681 encounters classified as temporarily deferred, 86.70% did not return to donate within the observation window. In a multinomial regression restricted to this subgroup, donation frequency before deferral was the strongest correlate of return (OR 21.02, 95% CI 14.94–29.58 for three or more subsequent donations among donors with ≥3 prior donations vs. none), and deferral for abnormal vital signs was also associated with higher odds of return (OR range 3.55–4.60, all *p* < 0.001). **Conclusions**: Nearly one-third of blood donor encounters at this tertiary center resulted in deferral, with abnormal vital signs and TTID screening reactivity as the predominant reasons. The low re-engagement rate (13.30%) among temporarily deferred donors underscores a need for structured follow-up programs, particularly for first-time donors, for whom an established donation history was the strongest predictor of return. Sex-specific interventions—targeting hemoglobin optimization in female donors and cardiovascular risk factor awareness in male donors—are warranted to reduce the deferral burden and safeguard the blood supply.

## 1. Introduction

Blood transfusions are among the most important life-saving medical interventions, forming an indispensable component of surgical care, hematological malignancy management, obstetric emergencies, and solid-organ transplantation. According to the World Health Organization (WHO), over 118 million blood donations are collected worldwide annually. However, demand continues to outstrip supply, particularly in low- and middle-income countries where the gap between clinical need and blood availability can be life-threatening. The safety and adequacy of blood products rely on a voluntary donor pool with appropriate pre-donation health screening [1].

Donor deferral is defined as the temporary, indefinite, or permanent exclusion of a prospective donor from blood donation based on failure to meet established eligibility criteria. There are three types of donor deferral: (i) Temporary Deferral, where the individual is excluded from donating blood for a set duration, after which their eligibility status is reassessed; (ii) Indeterminate (Indefinite) Deferral, where it becomes impossible to estimate the period of deferral until changes take place in either the donor’s health or the guidelines that are to be followed; and (iii) Permanent Deferral, where the individual is excluded for life [2]. These decisions are guided by national and international standards, including criteria set by the Saudi Food and Drug Authority (SFDA), the Central Board for Accreditation of Healthcare Institutions (CBAHI), the Association for the Advancement of Blood and Biotherapies (AABB), and the College of American Pathologists (CAP) [3,4].

Deferral rates vary substantially across regions and healthcare settings. Published rates range from approximately 5% in India [5,6] to nearly 19–31% in Iran, Malaysia, and parts of the Arabian Peninsula [7,8,9], reflecting differences in donor demographics, eligibility thresholds, screening technology, and disease endemicity. Direct comparison across these studies should be made cautiously, as deferral definitions differ: some studies capture only pre-donation deferral, whereas others, including the present study, also include post-donation deferral arising from infectious-disease screening reactivity. In Saudi Arabia, blood donation is primarily motivated by religious altruism and familial obligation, while barriers include needle phobia, health concerns, and the absence of active recruitment [10,11]. Earlier studies from Dhahran and Jeddah report that medication use, low hemoglobin, and abnormal vital signs constitute the principal pre-donation deferral causes [8,12].

Despite the clinical importance of deferral—both to individual donors and to the overall blood supply—data from large tertiary centers in Saudi Arabia remain scarce. Moreover, the downstream implications of deferral for donor retention have not been sufficiently examined. Deferred donors represent a unique population who, despite initial willingness to donate, experience exclusion that may diminish their intention to give again. Investigating post-deferral return behavior is therefore critical to developing effective donor retention programs.

King Fahad Medical City (KFMC), located in Riyadh, is among the biggest government-run hospital complexes in the Kingdom of Saudi Arabia. The organization comprises four specialty hospitals, a centralized blood bank, and a donor facility within a single unit. Its catchment provides large-centre data on donation deferral epidemiology in the capital region.

The present study therefore aimed to: (1) determine the overall deferral rate and its distribution across demographic strata (sex, age group, body weight, and nationality); (2) identify the most common deferral reasons overall and stratified by sex; (3) characterise deferral type (temporary, permanent, or indeterminate) and its relationship to pre-deferral donation history; and (4) quantify and identify independent predictors of post-deferral donation frequency among temporarily deferred donors, using retrospective electronic records from a two-year study period (2023–2024).

## 2. Materials and Methods

### 2.1. Study Design and Setting

This was a retrospective observational study conducted at the Blood Donation Centre of King Fahad Medical City (KFMC), Riyadh, Saudi Arabia. KFMC is a quaternary referral institution comprising the Main Hospital, Women’s Specialist Hospital, Children’s Hospital, and Rehabilitation Hospital, served by a single centralized laboratory and transfusion services unit. Donor assessment equipment included the CompoLab™ Fresenius Kabi Hemoglobin Analyzer (Fresenius Kabi AG, Bad Homburg, Germany) (using a cut-off of 12.5 g/dL for female donors and 13.5 g/dL for male donors) and the Abbott Cell-Dyn Emerald Hematology Analyzer (Abbo Laboratories, Abbott Park, IL, USA) (platelet count cut-off 150 × 10^9^/L for apheresis donors).

### 2.2. Study Period and Population

The study covered all voluntary blood donor encounters registered at KFMC from 1 January 2023 to 31 December 2024. The KFMC blood bank information system enforces a mandatory data-entry protocol requiring that all core variables (demographics, deferral classification, deferral reason, and donation frequency) be populated before a record can be closed; accordingly, no encounters were excluded from the analysis owing to missing data on these core variables. The final dataset comprised 60,290 donor encounters; the source records did not include a unique donor identifier, so whether these represent 60,290 distinct individuals or fewer individuals with repeat encounters could not be determined from the available data (see Section 4.4). All results are therefore presented at the encounter level and should be interpreted accordingly.

### 2.3. Donor Eligibility Criteria

Donor eligibility at KFMC follows protocols conforming to SFDA, CBAHI, AABB, and CAP standards. The principal pre-donation eligibility thresholds are: body weight ≥ 50 kg; body temperature ≤ 37.6 °C; hemoglobin (Hb) ≥ 13.5 g/dL for allogeneic male donors or ≥12.5 g/dL for allogeneic female donors; pulse rate 60–100 beats per minute; systolic blood pressure 90–180 mmHg; and diastolic blood pressure 50–100 mmHg. Additional criteria encompass medication history, infectious disease history, recent surgical or dental procedures, travel history to endemic disease zones, and behavioral risk factors for transfusion-transmitted infectious diseases (TTIDs).

### 2.4. Data Collection and Variables

Donor records were extracted from the Hematos IIG Medinfo International Hemoservice (version 5.2) blood bank information system ( GPI Group, Nice, France) into a structured Microsoft Excel spreadsheet. The following variables were recorded for each donor encounter:

(a) Demographics: sex, age group (≤30 years; 31–45 years; >45 years), body weight category (<50 kg; 50–60 kg; >60 kg), and nationality (Saudi/non-Saudi).

(b) Deferral classification: temporary (T), permanent (D), or indeterminate (I), as entered by the blood bank physician at the time of assessment; operational definitions of these categories are given in Section 1. In the KFMC system, “indeterminate” deferral is assigned in situations where the deferral duration cannot be determined at the time of assessment, including: donors requiring further evaluation for newly diagnosed medical conditions, donors with reactive screening results awaiting confirmatory testing, donors with incomplete clinical records requiring additional information, and donors with travel history to regions with emerging infectious disease risks where the deferral period cannot be specified until updated guidelines are issued.

(c) Deferral reason: a single primary standardized category recorded per encounter in the source system, including abnormal vital signs (high/low blood pressure, tachycardia/bradycardia); post-donation TTID screening reactivity (encompassing HBsAg, anti-HBc, anti-HCV, anti-HIV-1/2, anti-HTLV-I/II, HIV-1 RNA, HCV RNA, HBV DNA, serological syphilis testing, malaria testing, and bacterial detection for platelet units); low Hb; deferred medication use; post-donation blood group discrepancy or unexpected red-cell antibody; deferred disease; undetectable veins; underweight; physician-assessed probable risk (applied where the blood bank physician identified a risk factor for deferral not captured by the other standardized categories); incomplete inter-donation interval; self-deferral; travel history; surgical procedure; behavioral risk; under/over age (outside the 17–65-year range); low platelet count (apheresis); and insufficient quantity. (d) Donation frequency before deferral: none, once, twice, or three times or more. (e) Donation frequency after temporary deferral: within the observation window (follow-up range 2 to 26 months post-deferral), categorized as none, once, twice, or three times or more; the source data provided a single study-level extraction date rather than an individual deferral date for each encounter, and temporary deferral durations in the source data were recorded as eligibility dates only for the subset of donors who subsequently attended (i.e., date of return), with no individual deferral date recorded for non-returning donors. Consequently, donation frequency after temporary deferral was analyzed using fixed categories over a variable 2–26 month observation window rather than a formal time-to-event (Kaplan–Meier/Cox) analysis; donors deferred later in the study period had less opportunity to return than those deferred earlier, which may lead this study to underestimate the true return rate (see Section 4.4).

### 2.5. Statistical Analysis

Descriptive statistics are reported as frequencies and percentages (*n*/*N*, %). Associations between categorical variables were evaluated using the Pearson Chi-square test or, where any expected cell frequency was <5, Fisher’s exact test. Statistical significance was set at *p* < 0.05 (two-tailed), and *p*-values below 0.001 are reported as *p* < 0.001. A multinomial logistic regression model was constructed for donation frequency after temporary deferral (none/once/twice/three or more), using ‘none’ as the reference category, restricted to the subset of encounters with deferral status = Temporary (*n* = 10,681), since return donation is not a meaningful outcome for permanently or indeterminately deferred encounters. Age group and weight category were entered as categorical predictors with explicit reference levels (age ≤ 30 years; weight > 60 kg); the weight variable’s < 50 kg and 50–60 kg categories were combined into a single ≤60 kg stratum because of very few observations in the <50 kg group within the temporarily deferred subset. Independent variables entered into the model were sex, age group, weight category, presence of abnormal vital signs as the deferral reason, and donation frequency before deferral. Self-deferral, surgical procedure, and incomplete inter-donation interval were each recorded in fewer than 35 of the 10,681 temporarily deferred encounters and were evaluated separately via Fisher’s exact test rather than included in the multivariable model, because each had zero observed encounters in at least one outcome category, precluding stable maximum-likelihood estimation. Results are expressed as odds ratios (OR) with 95% confidence intervals (CI). No adjustment for multiple comparisons or for possible within-donor clustering was applied, given the exploratory nature of the analysis and the absence of a donor-level identifier in the source data. All analyses were performed in IBM SPSS Statistics, version 25 (IBM Corp., Armonk, NY, USA).

### 2.6. Ethical Considerations

The study was approved by the Institutional Review Board (IRB) of King Fahad Medical City (KFMC) under reference number 25-009 on 19 January 2025. As this was a retrospective review of anonymised administrative records, individual informed consent was waived by the IRB in accordance with applicable institutional and national guidelines. All data were handled in strict accordance with confidentiality requirements; no personally identifiable information was included in the analysis dataset. The study was conducted in accordance with the Declaration of Helsinki.

## 3. Results

### 3.1. Demographic Characteristics of the Donor Population

Between 2023 and 2024, a total of 60,290 volunteer blood donor encounters were registered at King Fahad Medical City. Of these, 18,458 (30.6%) resulted in deferral. The deferred cohort was predominantly male (86.4% vs. 13.6% female). Stratification by age showed that nearly half of the deferred encounters (47.3%) involved donors between 31 and 45 years old, while 32.6% involved donors 30 years of age or younger, and 20.1% involved donors older than 45. Additionally, the vast majority of deferred encounters (95.3%) involved donors weighing more than 60 kg. Demographically, Saudi nationals comprised 61.0% of the deferred group, with non-Saudi nationals accounting for the remaining 39.0%. The characteristics of the deferred cohort are summarized in Table 1.

### 3.2. Deferral Status and Major Causes

The main reasons for deferral across the study population were driven by physiological metrics and infectious disease screening. The most frequent reason was abnormal vital signs (39.4%)—encompassing hyper- or hypotension, tachycardia, and bradycardia. This was followed by reactive screening for transfusion-transmitted infectious diseases (TTIDs) post-donation, which accounted for 35.6% of deferrals (representing initial screening-assay reactivity rather than confirmed infection). Other reasons included low hemoglobin (Hb) levels (14.0%), the use of deferrable medications (4.0%), and discrepancies in blood grouping or the presence of unexpected antibodies post-donation (2.3%). Deferral statuses were classified into three primary categories based on duration: temporary (57.9%), indeterminate (29.9%), and permanent (12.2%) (Table 2).

### 3.3. Sex-Specific Deferral Trends

Analysis of the deferred cohort revealed distinct patterns between sexes. For male donors, the leading reason for deferral was abnormal vital signs, followed by post-donation reactive screening for transfusion-transmitted infectious diseases (TTIDs). Low hemoglobin (Hb) levels were the third most common reason, with the use of deferred medications being the least frequent. In contrast, female donors showed a different pattern: low Hb levels were the leading reason for deferral, followed by abnormal vital signs. Post-donation reactive TTID screening ranked third, and the use of deferred medications was least frequent (Table 3).

### 3.4. Donation Frequency Pre- and Post-Deferral

This study also evaluated the prior donation history of the deferred individuals. The highest proportion (41.2%) was among individuals that had never donated blood prior to their deferral. Donors who had donated once previously constituted 39.5%, those with two prior donations accounted for 7.6%, and those with three or more prior donations accounted for 11.8%. Among the 10,681 encounters classified as temporarily deferred, within the available observation window (which varied from 2 to 26 months), the rate of return was low: 86.70% did not return for any subsequent donation. Only 8.20% returned to donate once, 2.61% returned twice, and 2.49% returned three or more times. Because donors deferred later in the study period had less opportunity to return than those deferred earlier, this estimate should be interpreted as a descriptive measure over a variable window rather than a fixed follow-up return proportion (Table 4).

### 3.5. Predictors of Return Following Temporary Deferral

A multinomial logistic regression was fitted for donation frequency after temporary deferral (once, twice, or three or more times vs. none), restricted to the 10,681 encounters classified as temporarily deferred (Table 5). Male donors had modestly higher odds of returning once (OR 1.35, 95% CI 1.03–1.77, *p* = 0.031) and twice (OR 1.88, 95% CI 1.05–3.36, *p* = 0.034) than female donors, though not of returning three or more times (*p* = 0.194). Donors weighing ≤60 kg had higher odds of returning once than donors weighing >60 kg (OR 1.77, 95% CI 1.25–2.49, *p* = 0.001). Age group was not independently associated with return frequency once donation history before deferral was included in the model (all *p* > 0.09). Deferral for abnormal vital signs was consistently associated with higher odds of return across all three frequency categories (OR range 3.55–4.60, all *p* < 0.001). The strongest association was with donation frequency before deferral: donors with three or more prior donations had markedly higher odds of returning once (OR 2.72), twice (OR 10.22), and three or more times (OR 21.02) than first-time donors, all *p* < 0.001. Self-deferral, surgical procedure, and incomplete inter-donation interval were each recorded in fewer than 35 encounters and could not be reliably estimated within the multivariable model; unadjusted Fisher’s exact tests for these reasons against any subsequent donation were not statistically significant (self-deferral, *p* = 0.596; surgical procedure, *p* = 0.505; incomplete inter-donation interval, *p* = 1.000). These tests are presented for completeness but, because of small cell counts and the absence of covariate adjustment, should not be interpreted as definitive evidence of the absence of association.

## 4. Discussion

### 4.1. Demographic Characteristics and Divergence from Global Deferral Trends

Our study reveals an overall deferral rate of 30.6% within the KFMC cohort, driven primarily by abnormal vital signs and post-donation infectious-disease screening reactivity. This deferral rate is similar to that reported in Iran (30.9%) and higher than rates reported in Kenya (21.6%), Malaysia (18.8%), Nepal (9.5%), and two distinct cohorts in India (5.12% and 5.84%) [6,7,9,13,14,15]; these comparisons should be interpreted cautiously, as our deferral definition includes post-donation TTID screening reactivity, whereas several comparator studies capture only pre-donation deferral, which would tend to make our reported rate appear higher for reasons unrelated to true underlying donor eligibility. All comparisons in the present discussion are made at the encounter level and should be interpreted descriptively, as the absence of a donor-level identifier precludes donor-level inference. Furthermore, our demographic distribution differs from established global norms. While international cohorts typically identify younger individuals as the primary deferred demographic, such as 17–30 years in Shiraz (39.5%), 17–25 years in South India (42.92%), and 17–20 years in Dubai (35%) [6,7,16], the highest concentration of deferrals in our center occurred among donors aged 31 to 45 years (47.3%). Despite this age variation, the proportion of first-time donors among deferred encounters (41.2%) closely aligns with international trends documented in Malaysia (53.3%), Shiraz (48.2%), and South India (36.17%), consistent with a common pattern of higher deferral among first-time donors [6,7,9].

Temporary deferrals comprised the majority of the current cohort (57.9%). Such a proportion is comparable to the 58.04% temporary deferral rate observed in India, but lower than the 91.6% rate documented in Malaysia [5,9]. The overall clinical profile of deferral in this study is defined by three leading reasons: abnormal vital signs (39.4%), post-donation TTID screening reactivity (35.6%), and low hemoglobin (Hb) levels (14.0%). Notably, the high incidence of abnormal vital signs exceeds the 12.6% reported in Malaysia, though it more closely aligns with the 29.1% reported regionally in Dhahran [8,9]. The 35.6% post-donation TTID screening reactivity rate is markedly higher than the 0.5% rate documented in Malaysia [8,9]; this figure represents initial screening-assay reactivity rather than confirmed infection, and as marker-level and confirmatory-testing data were not available for this analysis, it should not be directly compared with confirmed-infection rates elsewhere. Conversely, studies from Iran report that deferrals are predominantly driven by HIV or hepatitis risk factors (43.70%) and underlying diseases (31.90%) [5,6,7,9,13]. Finally, the 14.0% deferral rate for low hemoglobin is similar to the 14.8% rate observed in Dhahran and reflects existing literature demonstrating that low hemoglobin is frequently a leading cause of deferral worldwide, driving 51.86% of deferrals in Kenya and 38.8% in Malaysia [8,9,13].

Stratification by sex revealed distinct patterns of deferral. Within the male cohort, exclusions were driven mainly by abnormal vital signs (40.00%) and post-donation TTID screening reactivity (39.10%), followed by low Hb levels (9.90%). This contrasts with male deferral patterns observed elsewhere; for example, clinical profiles in India are primarily driven by alcohol consumption, recent donations, and fever, while upper respiratory tract infections and high blood pressure are leading causes in Malaysia [5,7,9]. In Shiraz, HIV or hepatitis risk factors and underlying diseases predominate among male deferrals [5,7,9]. Conversely, the female deferral profile in our study aligns more closely with global norms, with low Hb (40.50%) constituting the leading reason, followed by abnormal vital signs (35.50%) and post-donation TTID screening reactivity (12.90%). The predominance of anemia-related deferrals in females is an established global trend, consistent with data from Malaysia (34.6%) and India [5,7,9]. This is distinct from cohorts in Iran, where underlying diseases (60.40%) and general non-eligible conditions (22.55%) predominate among female deferrals rather than hematological parameters [5,7,9].

### 4.2. Post Deferral Donor Behavior and Retention Challenges

A key finding of this study, interpreted descriptively over the available observation window, is the low re-engagement rate among temporarily deferred donors: 86.70% did not return for a subsequent donation within the variable observation window (2–26 months), while 13.30% returned at least once. This magnitude of non-return poses a substantial risk to the stability of the local blood supply chain, though the variable follow-up window means this should be interpreted as a descriptive estimate rather than a fixed-period return proportion. Prior literature indicates that a temporary deferral can act as a barrier to future donation, and that some temporarily deferred donors do not subsequently return, effectively becoming lost to the active donor pool despite their deferral not being permanent [17,18]. A deferral may disrupt the motivation to donate by inducing feelings of rejection, confusion, or health-related concern, effects that have been reported to be more pronounced among donors without an established donation routine [19].

Our multinomial regression analysis also examined how the reason for deferral was associated with subsequent donor behavior. Donors deferred for abnormal vital signs showed a higher likelihood of returning to donate across all frequency categories (OR range 3.55–4.60) compared with donors deferred for other reasons. This is consistent with findings elsewhere suggesting that donors may perceive physiological safety checks, such as blood pressure or pulse readings, as transient and modifiable [19]. By contrast, donors weighing more than 60 kg showed a lower likelihood of a single return donation than lighter donors (OR 1.77 for ≤60 kg vs. >60 kg), an association whose underlying mechanism was not directly measured in this study.

Age group was not independently associated with post-deferral return frequency once donation history before deferral was included in the model (all *p* > 0.09), suggesting that age associations reported in less-adjusted analyses elsewhere may in part reflect that older donors are more likely to have an established donation history, rather than age itself driving return behavior. Indeed, the strongest correlate of return in our cohort was donation frequency before deferral: donors with three or more prior donations had markedly higher odds of returning once (OR 2.72), twice (OR 10.22), and three or more times (OR 21.02) than first-time donors, all *p* < 0.001. This indicates that an already-established donation habit is far more predictive of post-deferral return than any single demographic factor measured here and identifies first-time donors who are deferred as the group at greatest risk of being lost to the donor pool entirely. These findings support a shift from broad donor recall strategies toward targeted retention interventions focused on reason for deferral and prior donation history.

### 4.3. Implications for Transfusion Services and Targeted Interventions

The distinct reason-specific and demographic patterns of deferral identified in this study suggest a shift from universal donor recruitment toward targeted and reason-specific retention strategies. Evidence from donor management literature suggests that the psychological friction of a temporary deferral can be lowered through structured communication [20]. Transfusion services could adopt automated and personalized digital outreach, such as SMS reminders, mobile application notifications, or targeted emails, timed to align with the expiration of the donor’s temporary deferral period. Such active recall systems have been associated with an increased probability of a return donation by reducing the burden of eligibility tracking on the donor [21].

Moreover, the marked sex-specific patterns of deferral reasons in our cohort suggest a role for tailored clinical interventions. Among female donors, low hemoglobin levels constituted the leading reason for deferral (40.50%). Blood donation centers could integrate proactive nutritional education and iron-related counselling at the point of deferral; providing female donors with structured educational materials on dietary iron intake, or protocols for iron supplementation following whole blood donation where clinically appropriate, may help support hemoglobin recovery and future eligibility [22].

Conversely, the predominance of abnormal vital signs among deferred male donors (40.00%) highlights an opportunity for blood donation centers to function as public health screening checkpoints. Because male donors deferred for vital sign abnormalities showed a relatively higher likelihood of returning for later donations, transfusion centers could leverage this engagement by providing brief cardiovascular health messaging at the time of deferral, for example advising the donor to monitor their blood pressure or consult a primary care physician. This may reframe the deferral from a rejection to a health-service touchpoint, and could contribute to earlier detection of asymptomatic hypertension within the broader community [23,24]. Implementing these reason-specific and supportive interventions may help transfusion services safeguard the blood supply while supporting the longitudinal health of their donor base.

### 4.4. Strengths and Limitations

This study has several strengths. It uses a large, comprehensive dataset comprising 60,290 registered donor encounters over a two-year period, providing large-centre data on blood donation dynamics at a major quaternary referral centre. Furthermore, moving beyond simple descriptive statistics, the application of a correctly scoped multinomial logistic regression to examine correlates of post-deferral donation frequency, adjusted for donation history before deferral, adds analytical depth. This methodological approach allows for a more detailed understanding of which physiological and demographic variables are associated with future donor behavior, offering insights for targeted recruitment and retention protocols. Additionally, this study addresses a gap in the literature by providing population-specific data from Saudi Arabia, a region previously underrepresented in global transfusion medicine research.

However, these findings must be interpreted in the context of several limitations. First, the study’s retrospective, single-center observational design at KFMC limits the generalizability of the results; the demographic profile and deferral reasons observed at a quaternary care facility in Riyadh may not fully reflect the dynamics present in rural centers, community blood drives, or non-tertiary facilities elsewhere. Second, the source records did not include a donor identifier, so whether some individuals contributed more than one encounter during the study period could not be assessed, and the chi-square/Fisher’s exact tests and multinomial regression used here do not account for potential within-donor correlation. The findings should therefore be interpreted at the encounter level rather than as donor-level behavior. Third, the accepted (non-deferred) donor population was not available for this analysis, so subgroup-specific deferral risk could not be calculated; the demographic findings reported here describe the composition of the deferred group rather than the risk of deferral itself. Fourth, the observation window for post-deferral return was variable (2–26 months) and could not be handled as a formal time-to-event analysis, because the source data did not provide an individual deferral date for non-returning donors and the eligibility period for return was recorded only for donors who subsequently attended (date of return). This means that donors deferred later in the study period had proportionally less opportunity to return. Consequently, the 86.70% non-return estimate and the multinomial regression results should be interpreted as descriptive encounter-level associations over a variable window, not as a formal time-to-return estimate. The true return proportion, measured over a fixed and adequately long follow-up period, could differ from the estimate reported here. Fifth, self-deferral, surgical procedure, and incomplete inter-donation interval could not be evaluated in the multivariable regression owing to small cell counts (each affecting fewer than 35 encounters); larger, ideally multi-center, samples would be needed to evaluate these reasons robustly. Sixth, post-donation TTID screening reactivity was recorded as a single aggregate category without marker-level or reactive-versus-confirmed breakdown, so this proportion should be interpreted as screening reactivity rather than confirmed infection. Furthermore, the source records did not distinguish between pre-donation and post-donation deferral at the point of data collection, so the TTID screening reactivity reported here reflects post-donation laboratory findings; as such, this group was not available for active follow-up or re-screening within the study window, and the deferral could not be resolved during the observation period. Seventh, our reliance on the blood bank’s electronic records restricted this analysis to quantitative variables; while the data show that 86.70% of temporarily deferred donors did not return, they lack the qualitative context to explain why. Future prospective, multi-center studies incorporating donor-level identifiers, individual deferral dates, marker-level TTID data, the full registered-donor population, and qualitative behavioral assessments are needed to build on these findings and further optimize donor retention strategies.

## 5. Conclusions

Blood donor deferral represents a significant challenge to transfusion services at KFMC, with an overall deferral rate of 30.6% driven largely by abnormal vital signs in men and low hemoglobin in women. Among donors classified as temporarily deferred, 86.70% did not return to donate again, highlighting a gap in donor management that threatens blood supply stability. An established donation habit before deferral was, by a wide margin, the strongest predictor of return afterward, identifying first-time donors who are deferred as the group at greatest risk of being lost to the donor pool.

Securing the future blood supply will likely require a shift from universal recruitment toward targeted, reason- and history-specific interventions that re-engage deferred individuals, including prioritized outreach to first-time donors who are deferred. However, because the data were analyzed at the encounter level, these conclusions should be interpreted descriptively and would benefit from validation in a donor-level dataset. Automated recall systems, nutritional counselling for female donors, and cardiovascular health screening opportunities for male donors represent promising strategies. Future research should employ prospective, multi-center designs incorporating donor-level identifiers, individual deferral dates, and qualitative assessments to further elucidate the barriers to donor return, ultimately optimizing retention strategies and safeguarding transfusion services.

## Figures and Tables

**Table 1:** Characteristics of the individuals who were deferred from blood donation.

Characteristics of the Deferred Donor Encounters (*n* = 18,458)			
	*n*/*N* (Deferred)	Percentage of Total Deferred Donors	Percentage of Total Registered Donors
Sex (Male/Female)			
Male	15,954/18,458	86.4%	26.46%
Female	2504/18,458	13.6%	4.15%
Age group (Years)			
≤30	6024/18,458	32.6%	10%
31–45	8730/18,458	47.3%	14.5%
>45	3704/18,458	20.1%	6.14%
Weight (Kg)			
<50	130/18,458	0.7%	0.2%
50–60	740/18,458	4.0%	1.22%
>60	17,588/18,458	95.3%	29.2%
Nationality			
Saudi	11,260/18,458	61.0%	18.7%
Non-Saudi	7198/18,458	39.0%	12%
Total	18,458/18,458	100%	30.6%

**Table 2:** Most common deferral reasons and deferral status.

Deferral Reasons (Descending)	*n*/*N*	Percentage of Total Deferred Donors	Percentage of Total Registered Donors
Abnormal vital signs	7273/18,458	39.4%	12%
Post-Donation TTID Screening Reactivity	6566/18,458	35.6%	11%
Low Hb	2591/18,458	14.0%	4.3%
Using Deferred Medication	740/18,458	4.0%	1.2%
Post Donation Blood Grouping Discrepancy/Red Cells Ab Screening Positive	420/18,458	2.3%	0.7%
Deferred Diseases	208/18,458	1.1%	0.3%
Undetectable veins	138/18,458	0.7%	0.2%
Underweight	130/18,458	0.7%	0.2%
Physician-assessed probable risk *	70/18,458	0.4%	0.1%
Incomplete inter-donation interval	56/18,458	0.3%	0.09%
Other	266/18,458	1.4%	0.4%
Total	18,458/18,458	100%	30.6%
Deferred Status (Descending)	*n*/*N*	Percentage of Total deferred	Percentage of Total Registered Donors
Temporary	10,681/18,458	57.9%	18%
Permanent	2260/18,458	12.2%	3.7%
Indeterminate	5517/18,458	29.9%	9.2%
Total	18,458/18,458	100%	30.6%

Note: * “Physician-assessed probable risk” refers to encounters where the blood bank physician identified a risk factor for deferral not captured by the other standardized categories (includes tattooing within the deferral window). “Other” aggregates all remaining low-frequency deferral reasons (each <35 encounters), including self-deferral, surgical procedure, behavioral deferral, insufficient quantity, travel history, under/over age, general unwellness, low platelet count for apheresis, contact with a TTID-infected person, recent vaccination, recent dental procedure, and recent delivery.

**Table 3:** Top ten deferral reasons among donors.

Top Ten Reasons for Deferral in Male Donors (Descending)	*n*/*N*	Percentage	Percentage of Total Registered Donors
Abnormal vital signs	6384/15,954	40.00%	10.6%
Post-Donation TTID Screening Reactivity	6242/15,954	39.10%	10.4%
Low Hb	1577/15,954	9.90%	2.6%
Using Deferred Medication	666/15,954	4.17%	1.1%
Post-Donation Blood Grouping Discrepancy/Red Cells Ab Screening Positive	378/15,954	2.40%	0.6%
Deferred diseases	184/15,954	1.20%	0.3%
Undetectable veins	99/15,954	0.60%	0.2%
Underweight	87/15,954	0.50%	0.14%
Physician-assessed probable risk	58/15,954	0.40%	0.09%
Incomplete inter-donation interval	54/15,954	0.30%	0.09%
Other	225/15,954	1.41%	0.4%
Total	15,954/15,954	100%	26.5%
Top ten reasons for deferral in female donors (Descending)	*n*/*N*	Percentage	Percentage of Total Registered Donors
Low Hb	1014/2504	40.50%	1.7%
Abnormal vital signs	889/2504	35.50%	1.5%
Post-Donation TTID Screening Reactivity	324/2504	12.90%	0.5%
Using Deferred Medication	74/2504	3.00%	0.12%
Underweight	43/2504	1.70%	0.07%
Post Donation Blood Grouping Discrepancy/Red Cells Ab Screening Positive	42/2504	1.70%	0.069%
Undetectable veins	39/2504	1.60%	0.06%
Deferred diseases	24/2504	1.00%	0.04%
Physician-assessed probable risk	12/2504	0.50%	0.02%
Incomplete inter-donation interval	2/2504	0.10%	0.003%
Other	41/2504	1.60%	0.07%
Total	2504/2504	100%	4.2%

**Table 4:** Frequency of donations before deferral and after temporary deferral.

Frequency of Donations Before Deferral (Descending)	*n*/*N*	Percentage	Percentage of Total Registered Donors
None	7596/18,458	41.2%	12.6%
Once	7283/18,458	39.5%	12.08%
Twice	1404/18,458	7.6%	2.3%
Three times or more	2175/18,458	11.8%	3.6%
Total	18,458/18,458	100%	30.6%
Frequency of donations after temporary deferral (restricted to temporarily deferred encounters, *n* = 10,681)	*n*/*N*	Percentage	Percentage of Total Registered Donors
None	9260/10,681	86.70%	15.36%
Once	876/10,681	8.20%	1.45%
Twice	279/10,681	2.61%	0.46%
Three times or more	266/10,681	2.49%	0.44%
Total	10,681/10,681	100%	17.72%

**Table 5:** Multinomial Regression for Post-Temporary Deferral Donation Frequency.

Predictor	Once vs. None		Twice vs. None		Three or More vs. None	
	OR (95% CI)	*p*-Value	OR (95% CI)	*p*-Value	OR (95% CI)	*p*-Value
Sex: Male (ref. Female)	1.35 (1.03–1.77)	0.031	1.88 (1.05–3.36)	0.034	1.44 (0.83–2.48)	0.194
Age 31–45 y (ref. ≤ 30 y)	1.15 (0.98–1.35)	0.096	1.14 (0.85–1.53)	0.392	0.86 (0.64–1.16)	0.332
Weight ≤ 60 kg (ref. > 60 kg)	1.77 (1.25–2.49)	0.001	1.10 (0.56–2.17)	0.773	1.28 (0.69–2.39)	0.431
Abnormal vital signs (deferral reason)	3.55 (2.94–4.28)	<0.001	3.79 (2.82–5.11)	<0.001	4.60 (3.37–6.27)	<0.001
Prior donations: Once (ref. None)	0.58 (0.47–0.71)	<0.001	0.97 (0.66–1.44)	0.885	1.39 (0.90–2.15)	0.140
Prior donations: Twice (ref. None)	1.80 (1.39–2.32)	<0.001	3.88 (2.54–5.94)	<0.001	4.16 (2.50–6.94)	<0.001
Prior donations: ≥3 (ref. None)	2.72 (2.21–3.36)	<0.001	10.22 (7.45–14.02)	<0.001	21.02 (14.94–29.58)	<0.001

## Data Availability

The data supporting the findings of this study are available from the corresponding author upon reasonable request, subject to institutional data-sharing policies.

## Abbreviations

The following abbreviations are used in this manuscript:

TTID	Transfusion-Transmitted Infectious Disease
SFDA	Saudi Food and Drug Authority
Hb	Hemoglobin
KFMC	King Fahad Medical City
IRB	Institutional Review Board
CBAHI	Central Board for Accreditation of Healthcare Institutions
AABB	Association for the Advancement of Blood and Biotherapies
CAP	College of American Pathologists
OR	Odds Ratio
CI	95% Confidence Interval
